# Word frequency cues word order in adults: cross-linguistic evidence

**DOI:** 10.3389/fpsyg.2013.00689

**Published:** 2013-10-02

**Authors:** Judit Gervain, Núria Sebastián-Gallés, Begoña Díaz, Itziar Laka, Reiko Mazuka, Naoto Yamane, Marina Nespor, Jacques Mehler

**Affiliations:** ^1^Laboratoire Psychologie de la Perception, Université Paris Descartes, Sorbonne Paris CitéParis, France; ^2^Laboratoire Psychologie de la Perception, CNRSParis, France; ^3^Department of Technology, Center for Brain and Cognition, Universitat Pompeu FabraBarcelona, Spain; ^4^Lingusitics and Basque Studies, Psycholingusitics Laboratory, University of the Basque Country (UPV/EHU)Vitoria-Gasteiz, Spain; ^5^Laboratory for Language Development, RIKEN Brain Science InstituteWako-shi, Japan; ^6^Department of Psychology and Neuroscience, Duke UniversityDurham, NC, USA; ^7^Language, Cognition and Development Lab, Cognitive Neuroscience Sector, SISSA - International School for Advanced StudiesTrieste, Italy

**Keywords:** language acquisition, speech perception, morphosyntax, cross-linguistic analysis, word frequency, corpus analysis, anchoring hypothesis

## Abstract

One universal feature of human languages is the division between grammatical functors and content words. From a learnability point of view, functors might provide entry points or anchors into the syntactic structure of utterances due to their high frequency. Despite its potentially universal scope, this hypothesis has not yet been tested on typologically different languages and on populations of different ages. Here we report a corpus study and an artificial grammar learning experiment testing the anchoring hypothesis in Basque, Japanese, French, and Italian adults. We show that adults are sensitive to the distribution of functors in their native language and use them when learning new linguistic material. However, compared to infants' performance on a similar task, adults exhibit a slightly different behavior, matching the frequency distributions of their native language more closely than infants do. This finding bears on the issue of the continuity of language learning mechanisms.

## Introduction

Speakers of English readily recognize “***Twas*** *brillig*, ***and the*** *slith**y*** *tove**s*** \ ***Did*** *gyre* ***and*** *gimble* ***in the*** *wabe* …,” the first lines of Lewis Carroll's *Jabberwocky* poem as having an English-like grammatical structure despite the absence of any meaning. This is a striking illustration of a universal feature of human languages: grammatical functors (set in bold in the quote) define and signal sentence structure, while content words carry meaning. Languages differ with respect to which universally available content or function word categories they instantiate and how they implement them, but the major divide between function and content words is present in all the world's languages (Fukui, 1986; Abney, 1987). Using a cross-linguistic perspective, the present paper investigates whether this feature of human languages contributes to their parsing and learning in adult speakers. The main goal of the paper is to show that the anchoring property of function words applies in typologically different languages and can be used even by adults, i.e., speakers with a full-blown linguistic competence.

Function words have been hypothesized to contribute to language learning in at least two ways. First, they often help categorize content words. In English, for instance, nouns are typically preceded by determiners such as *a, the, some*, etc., whereas verbs are often preceded by auxiliaries, such as *have, is*, etc. and they take suffixes like -*ing*, -*ed*, etc. Formally described first by structuralist linguists (Bloomfield, 1933; Saussure et al., 1983), this role of functors has been central to formalist as well as statistical theories of language, and numerous behavioral and computational studies have established its psychological relevance (e.g., Thorne, 1968; Morgan et al., 1996; Redington et al., 1998; Shi et al., 1998, 1999; Mintz, 2002; Shi and Werker, 2003; Shi et al., 2006).

Second, functors have been assumed to cue rules and increase the learnability of structural generalizations in language. Intuitively, functors, due to their high frequency, act as anchor points with respect to which the structural roles and sequential positions of other constituents can be encoded and remembered. This hypothesis has been explored in a number of artificial grammar learning studies, asking whether (artificial) languages with and without the functor/content word distinction show different degrees of learnability (Braine, 1966; Green, 1979; Morgan and Newport, 1981; Mori and Moeser, 1983; Morgan et al., 1987; Valian and Coulson, 1988; Valian and Levitt, 1996; Wang et al., 2011). These studies, discussed in detail below, confirm that functors contribute to the learnability of linguistic structure by serving as structural flags or anchor points for English-speaking participants.

However, functors are realized differently across languages, one important difference being their relative order with respect to content words. Whether functors serve as anchor points in languages with different configurations remains largely unexplored. A few recent studies with infants exposed to languages other than English (Höhle et al., 2001; Gervain et al., 2008; Hochmann et al., 2010) suggest that young learners of typologically diverse languages do use functors as entry points into language structure. However, the number of languages investigated remains limited (German, Italian, and Japanese). Further, it is also unknown whether the anchoring role of functors in these typologically different languages is present only at the beginning of the language acquisition process, or whether it is a strategy that even adults rely on when parsing novel linguistic material. Arguments have been put forth in the literature for both the continuity and the discontinuity of language learning and competence across the life span (Guasti, 2002; Santelmann et al., 2002).

The aim of the current study is, therefore, twofold. First, it seeks to investigate parsing strategies based on the distribution of frequent items, used by adult speakers of two pairs of typologically different languages: Basque and Japanese as well as Italian and French. French and Basque have never been investigated from this perspective in the literature before. Second, by testing adult speakers in a task adapted from one of the previous infant studies (Gervain et al., 2008), we also seek to explore the life span continuity of this learning strategy. The inclusion of Italian and Japanese participants will allow us to compare their performance with the Italian and Japanese infants tested in the previous study.

Below, we first provide a brief description of the relevant typological differences between Basque, Japanese, Italian, and French, highlighting the fact that Basque and Japanese are typically characterized as functor-final, whereas French and Italian are functor-initial languages. Afterwards, we discuss the anchoring hypothesis in detail, reviewing the relevant behavioral studies, and whether such a language learning mechanism might be employed across the life span. We then report a corpus study showing that functors do indeed act as utterance-final anchors in Basque and Japanese and utterance-initial in Italian and French in actual language use. This is followed by an artificial grammar study, which shows that adult native speakers use functors as entry points to the structure of an unknown language and they do so using the word orders and frequency distributions characteristic of their native language. When comparing the performance of the adults in our study and that of the infants in the previous study (Gervain et al., 2008), we find both important similarities and differences. The discussion of their theoretical implications closes the paper.

### Functors and content words in the world's languages

The distinction between grammatical functors and semantically loaded content words is universal (Fukui, 1986; Abney, 1987; Morgan et al., 1996). However, the world's languages show systematic differences in the way they use functors and position them with respect to content words. English, French, and Italian, for instance, use prepositions in front of nouns (e.g., English: *on the table*). By contrast, Japanese, Basque, and Turkish have postpositions [e.g., Japanese: *Kobe ni* Kobe to “to Kobe (a city in Japan)”]. The relative order of functors and content words correlates with other word order phenomena (Greenberg, 1963; Dryer, 1992). Languages with prepositions typically use a Verb-Object (VO) basic word order (e.g., English: *eat an apple*), whereas postpositional languages are usually Object-Verb (OV) languages (e.g., Japanese: *ringo-wo taberu* apple.acc eat). The order of constituents in other phrase types (e.g., embedded clauses, possessives, etc.) correlates with basic word order (Greenberg, 1963; Dryer, 1992). Determining the relative order of functors and content words might thus be a powerful cue to a large number of syntactic structures in a language.

Another important typological difference between languages is whether they realize functors as bound or free morphemes. Bound morphemes are morphologically dependent on, i.e., attached to, other words (e.g., *read-ing*), whereas free morphemes are independent words (e.g., *to read*). Functors that appear as free morphemes in VO languages often surface as bound morphemes in OV languages (e.g., prepositions vs. suffixes, respectively; English: *on the table* vs. Basque: *mahai-a-n* table-the-on). In fact, a systematic relationship exists between word order, on the one hand, and the bound vs. free nature and the position of functors, on the other (Greenberg, 1963; Dryer, 1992). When functors are realized as bound morphemes, there is a general tendency in the world's languages to realize them as suffixes, not prefixes. However, this general preference for suffixes is modulated by word order: suffixation is predominant in OV languages, but it is also common in VO languages, whereas prefixes are rare in OV languages and exclusive prefixation only exists in VO languages, never in OV languages.

Since word order varies across languages, young infants face the task of having to learn this morphosyntactic property from the speech input when acquiring their native language. Babies seem to accomplish this at an early age. In fact, their first multiword utterances (toward the end of the second year of life) follow the basic word order of the target language (Brown, 1973). Does the anchoring function of grammatical markers play a role?

Recently, Gervain et al. (2008) reported that infants as young as 7 months of age are able to form a rudimentary representation of word order on the basis of word frequencies. Given the correlation of the relative position of functors and content words with other word order phenomena within a language (Dryer, 1992), keeping track of the relative order of frequent and infrequent words, i.e., functors and content words, might provide infants with a heuristic cue to a rudimentary representation of basic word order. This is exactly what Gervain et al. (2008) found. Using an artificial grammar learning task, they showed that 8-month-old Italian and Japanese infants had opposite expectations about the relative order of frequent and infrequent words, mirroring the opposite word orders of their native languages, well before they start to talk^1^. Italian infants preferred the test items with frequent-infrequent (FI) order, while Japanese looked longer at the infrequent-frequent (IF) order. This suggests that the distribution of functors might indeed contribute to language learning.

### Functors contribute to the learnability of language

Braine (1966) was one of the first to study how frequent or constant marker elements influence grammar learning. Linear order is a fundamental aspect of natural languages. However, typically what is important in grammar is not the exact ordinal position of a word in a sequence, but the position of constituents with respect to each other (Chomsky, 1957). For instance in English *wh*-questions, the auxiliary follows the *wh*-phrase irrespectively of its length. Consequently, it may occupy different ordinal positions within different sentences, but always the same position with respect to the *wh*-phrase (e.g., [*Where*]*are they*? [*How many*] *are they*?, but not ^*^How are many they?). Therefore, it is important to know what mechanisms enable humans to learn languages on the basis of information about underlying (non-adjacent) dependencies rather than ordinal position. Braine (1966) tested this in 9–10-year-old children, giving them artificial grammar learning tasks in which success depended on learning the positions of non-frequent variable tokens (“content words”) with respect to constant marker elements (“function words”). The positions to be learnt could be immediately adjacent to or one position removed from the marker element (as in the structure fPQ, where f is a marker, P and Q are content words—a natural language example would be: *the blue car*). The results suggested that participants readily learned both relative positions. This, as the author pointed out, was a necessary prerequisite for natural language acquisition. Indeed, Braine (1963a) observed that functors often play the role of “pivot” during young infants' two-word stage production. These utterances often contain a closed-class word, which is productively combined with an open-class word (*my daddy, my mommy, my milk* …). The early appearance of some functors in language production points to their role in the acquisition of grammatical structure^2^.

Green (1979) investigated the importance of the reliability of functors as markers. In a first experiment, he presented three different grammars to three groups of adult participants. The first group saw well-formed strings from a grammar containing functional markers and content words, which co-occurred in a systematic way (“effective markers”). The second group was familiarized with a grammar having markers and content words, which co-occurred randomly (“useless markers”). The third group was presented with a grammar having no markers (“no markers”). The author found that there was some learning in all three conditions, but learners of “effectively marked” grammars performed significantly better than participants in the other two conditions. Green (1979) summarized these findings in the “marker hypothesis,” which has the following three tenets. First, in all learnable languages, there is a small set of words or morphemes (i.e., function words), the “markers,” each of which is associated with one or, at most, a few syntactic constructions/categories. Second, sentences are easier to parse when they contain markers. Third, a language without markers would be very difficult or impossible to parse and, hence, to learn^3^.

Morgan et al. (1987) conducted similar experiments, comparing learning in artificial grammars that had (i) no markers, (ii) inconsistent markers, or (iii) consistent markers. They focused mainly on how, if at all, markers help learners discover the hierarchical phrase structure of the input. Importantly, they tested free and bound functors, i.e., function words and grammatical suffixes, separately. In the experiment that tested free function words, three grammars were used. One (“no markers”) contained only content words, and no function words. A second (“inconsistent markers”) used both function words and content words, but function words appeared randomly between content words. A third (“consistent markers”) had both function words and content words, in such a way that function words indicated phrase boundaries. Apart from the functors, all three grammars were generated by the same phrase structure rules. Participants learned the linear order and sequential co-occurrence patterns of words in all conditions. However, those in the consistent markers condition performed better than the others. Moreover, only they succeeded in a subsequent constituency test. The second experiment tested the same three grammars but using bound morphemes instead of free markers. The results were similar to the ones obtained before. Morgan et al. (1987) concluded that markers, both free and bound, provided efficient cues to hierarchical phrase structure.

Some of the above studies tested children; others tested adults. Can we consider language learning in a lifespan perspective?

### The continuity of grammar learning mechanisms across life

The question whether linguistic abilities are best characterized by continuity or discontinuity across life has been of considerable theoretical importance for language acquisition research and linguistic theory (e.g., Weissenborn et al., 1992; Guasti, 2002). The related issue of a critical or sensitive period for language learning and its implications for neural plasticity have also attracted much attention (for a recent review, see Fava et al., 2011).

Younger learners clearly outperform older ones in several domains of language learning, especially those related to the sound patterns and morphosyntactic regularities of language, while vocabulary is less challenging to learn even at an older age. It is well-established that native phonological perception and production is very difficult to achieve if the first exposure to a language occurs after early childhood (Dupoux et al., 1997; Pallier et al., 1997; Sebastian-Galles and Soto-Faraco, 1999; Best and McRoberts, 2003). In morphosyntax, adults' disadvantage is somewhat less marked, although fully native-like proficiency is still hard to achieve (Johnson and Newport, 1989; Long, 1990). However, even in the case of comparable performance, infant and adult populations might not rely on the same learning mechanisms or linguistic competence to achieve a similar performance. Specifically, in the domain of morphosyntactic acquisition, the aspect of language relevant for our study, a series of studies by Newport and colleagues (Johnson and Newport, 1989; Newport, 1990; Goldowsky and Newport, 1993; Hudson Kam and Newport, 2005; Wonnacott et al., 2008; Hudson Kam and Newport, 2009) suggests that younger and older learners might use two basic learning mechanisms, memory-based/statistical learning and rule extraction, differently at least under some conditions. Captured by the “less is more” hypothesis, Newport (Newport, 1990; Goldowsky and Newport, 1993) argues that infants tend to rely more heavily on rule extraction and regularization, because their memory capacity, more limited than that of adults, prevents them from memorizing large data sets item by item. Given limited memory, the most efficient way to encode and learn a dataset is to extract regularities. Adults, not limited by memory constraints in the same way, might rely more on memorizing, item-based or statistical learning instead. Indeed, late learners of a language have been observed to memorize entire unanalyzed chunks or sequences, which young learners tend to decompose instead (e.g., for American Sign Language; Newport, 1984). Similarly, experiments testing how faithfully younger and older learners encode morphosyntactic properties that appear probabilistically or inconsistently in the input found that children were more likely to regularize inconsistencies, while adults only did so for the most inconsistent features (Hudson Kam and Newport, 2009). Taken together, these results suggest that both statistical learning and rule extraction are available to young and adult learners alike. However, the two age groups might employ these mechanisms somewhat differently, predicting both similarities and differences in how morphosyntax is learned at different ages.

### The current study

In the present study, we seek to extend the existing research on the role of frequent functors in parsing and leaning new linguistic material. Gervain et al.'s (2008) study tested one language per word order type. It remains to be determined whether the specificities of the two languages suffice to account for the results or whether they are generalizable to typologically similar languages. The first hypothesis we test in this study is that the frequency-based strategy will generalize to other languages. In addition, it remains unexplored whether adult speakers rely on the position of frequent words in their native language when segmenting novel linguistic input. The second hypothesis examined here is that adults might also use frequent words as anchor points when parsing new material, although they might rely on somewhat different mechanisms than infants.

The present paper thus examines these hypotheses in adult speakers of four languages representing the above discussed typological variants. Japanese, Basque, Italian, and French were selected. French is a functor-initial V(erb)–O(bject), Preposition–Noun language (e.g., *manger une pomme* eat an apple “eat an apple,” *sur la table* on the table “on the table”), similar to Italian in most morphosyntactic properties. By contrast, Basque, like Japanese, is a functor-final OV, Noun-Postposition language (e.g., *sagarra jan* apple eat “eat an apple,” *mahai-a-n* table-the-on “on the table”). However, unlike Japanese, Basque has postnominal determiners (e.g., *gizon-a* man-the “the man,” *lehendakari-a* president-the “the president”). This makes Basque even more consistently functor-final than Japanese, as most bound morpheme functors occupy final positions in syntactic phrases (Hualde and Ortiz de Urbina, 2003; de Rijk and de Coene, 2008). For free functors, no marked difference exists between the two languages.

While these linguistic descriptions hold at the grammatical level, from a learnability point of view it is important to show that they manifest themselves in a statistically reliable fashion in actual language use, serving as input for language learning. Therefore, our first aim is to show that the most frequent elements are indeed functors in all four languages and that they occupy phrase-initial positions in French and Italian, and phrase-final positions in Basque and Japanese.

## Study 1: A corpus study

In this analysis, we investigate whether the sequential position of functors in actual French, Italian, Basque, and Japanese corpora follows the distributions predicted by the anchoring hypothesis. Since functors typically constitute at least the 20–30 most frequent morphemes in a language, we operationalized this question by calculating the proportion of frequent item final and frequent item initial “phrases” in two privileged positions within utterances, i.e., their beginnings and ends. Utterance boundaries were chosen because they are identifiable via perceptual, i.e., non-grammatical, non-structural, cues (Aslin et al., 1996). Examining phrases within utterances would have been circular, as the anchoring hypothesis provides a bootstrapping strategy precisely to break into and bracket the internal syntactic structure of utterances. Utterance boundaries, by contrast, can be identified without any grammatical knowledge through prosodic and phonological cues.

### Materials and methods

#### Corpora

We used corpora from four languages. In Japanese, Italian, and French, transcriptions of actual speech directed to infants and young children were available. For Basque, currently no such database exists. We, therefore, used a variety of written sources, mainly texts extracted from newspapers as well as children's books.

The four languages we examined have different orthographic traditions. In Japanese, for example, postpositions are written as separate words, while in Basque, they are attached to the noun. To eliminate such differences, agglutinative affixes (i.e., elements of inflectional morphology attaching to the word stem, e.g., Basque *mendi-tik* mountain from) were encoded as separate morphemes^4^. Thus, the corpora were tagged and segmented into morphemes. This allowed us to take both free and bound functors into account, better reflecting adult speakers' full-fledged (but, of course, implicit) knowledge of their native grammar. The corpora were phonologically transcribed to provide a more realistic signal using phonotypical pronunciations in a semi-automated manner (Roach et al., 1996).

For each language, we collected 10 subcorpora from independent sources, i.e., different speakers or different texts, in order to have independent data points for each language, allowing statistical analysis. Each subcorpus in each language comprised 500 utterances, for a total of 5000 utterances per language. We relied on the original corpora for the definition of utterances. We counted as a single utterance whatever was transcribed as such in the original corpora. Equating the number of utterances per subcorpus and per language allowed us to better compare languages and to control for potential biases resulting from sample size and data sparsity, which might affect linguistic variability and frequency counts, at least for medium or low frequency linguistic features (Biber and Finegan, 1991; De Haan, 1992).

For Basque, we used 10 randomly chosen extracts from written sources (two newspapers and eight books) courtesy of The Basque Language Institute (http://www.ei.ehu.es/). Each subcorpus was 500 sentences long, thus this corpus comprised 5000 utterances. For Japanese, we made use of the corpus of infant-directed Japanese collected at the Laboratory of Language Development, Brain Science Institute, RIKEN (Mazuka et al., 2006). For our purposes, we extracted 500-utterance-long samples from 10 mothers' utterances addressed to their infants during free play or directed story-telling (using specific story books), but we excluded their conversations with adults, e.g., the experimenter. Our full corpus thus comprises 5000 utterances. For Italian, we used 500-utterance-long samples from the utterances of 10 adults from four Italian language subcorpora (Antelmi, 2004; Antinucci and Parisi, 1973; Cipriani et al., 1989; Tonelli et al., 1995; Salerni et al., 2007) of the CHILDES database (MacWhinney, 2000). The full corpus comprises 5000 utterances. For French, we used 500-utterance-long samples of the speech of 10 adults from the Paris subcorpus (Morgenstern and Parisse, 2007; Leroy et al., 2009; Morgenstern and Sekali, 2009) of the CHILDES database. The resulting full corpus consists of 5000 utterances.

#### Measures

We used the multiword utterances of the corpora to calculate how often frequent and infrequent items appear at phrase-initial and phrase-final positions at utterance boundaries. Single word utterances were discarded, as they are uninformative with respect to word order^5^. Frequent and infrequent items were defined as having a relative frequency of occurrence higher and lower, respectively, than a predefined threshold *T* = 0.01^6^. (Relative frequency of occurrence is the absolute frequency of occurrence of a given item normalized by the size of the corpus, allowing comparisons across corpora of different sizes.) In this frequency range, most items are closed-class morphemes in all four languages (e.g., Basque: *-ko* locative suffix, *du* “has.3sg.transitive”; Japanese: *chan* diminutive honorific to address children, *-wa* topic marker; Italian: *il* masculine definite article, *che* “what”; French: *est* “is,” *pas* negative particle). All other morphemes in the corpora were categorized as infrequent.

Using the frequent and infrequent categories as defined above, we calculated the percentages of the different possible orders at the boundaries of multiword utterances. We obtained these measures in the following way. We identified the first and the last two items of utterances, i.e., bimorphemic “phrases” at the left and right utterance boundaries. If the “phrase” had a [frequent item—infrequent item] order, it was counted as FI. Examples of FI phrases include è *rosso* is red “(it) is red,” *all*' *asilo* in-the daycare “in the daycare” [Italian, presented orthographically for ease of exposition]. If it had an [infrequent item—frequent item] structure, it was counted as IF. Examples of IF phrases include *zoritxar haren* misfortune his “his bad luck” [Basque]. “Phrases” where both morphemes were of the same category, i.e., [frequent item—frequent item] or [infrequent item—infrequent item] did not enter into the counts^7^.

For statistical purposes, we calculated the proportion of FI and IF utterances in each subcorpus in the above defined way, and conducted analyses of variance over the obtained datasets. We expect to find opposite word orders in Japanese and Basque, on the one hand, and Italian and French, on the other. We further predict a possible difference between two OV languages, as Basque has more functor-final phrases than Japanese. No such difference is expected between Italian and French.

### Results

**Figure 1:**
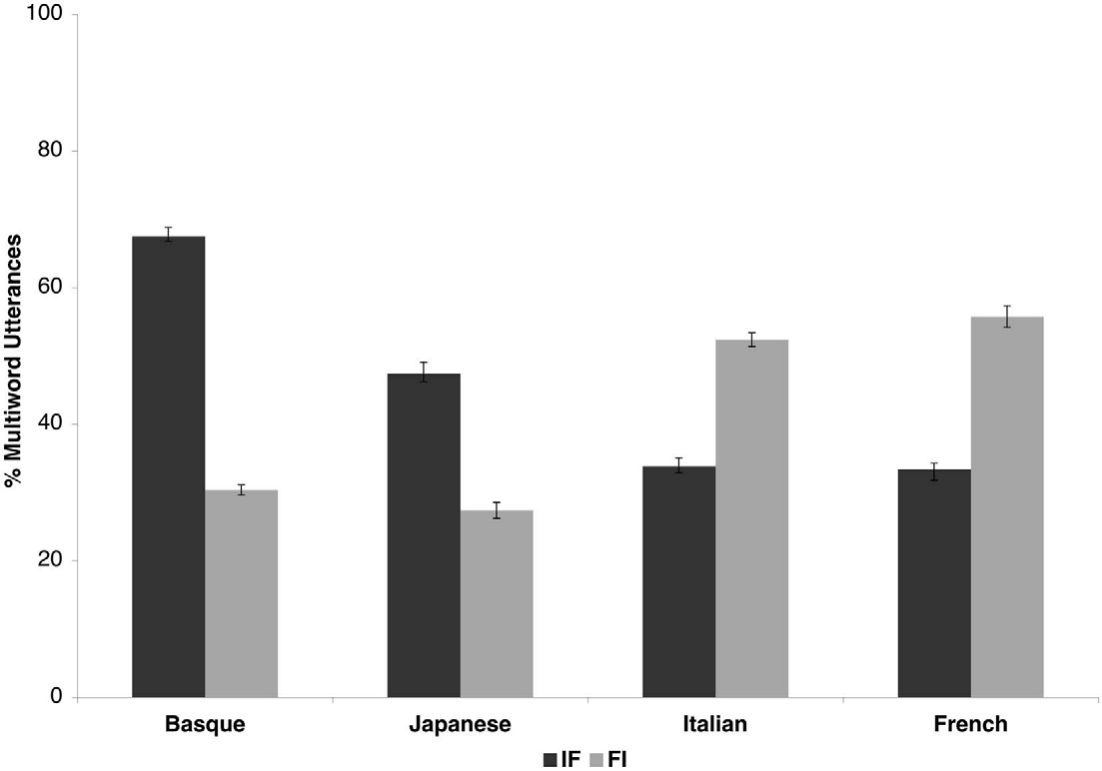
**The results of the corpus study**. The x-axis shows the four languages. The y-axis represents the percentage of FI (light gray bars) and IF (dark gray bars) phrases at the boundaries of multiword utterances in the four corpora. Note that the maximum value is 200%, as each utterance contributes two data points (one for the beginning, one for the end). Error bars represent the standard error of the mean.

Figure 1 presents the percentages of FI and IF utterances in the four languages. As expected, the OV languages (Japanese: 38.4% IF, 20.36% FI and Basque: 66.86% IF, 20.36% FI) and the VO languages (Italian: 39.64% IF, 60.16% FI, and French: 39.06% IF, 57.54% FI) show opposite patterns, the former having more IF utterances, the latter more FI ones. All the statistical analyses reported below were also carried out with the additional factor Position (sentence-initial/sentence-final), but as the factor had no significant main effect, nor did it enter into significant interactions in any of the analyses, data from the initial and final positions were pooled.

We carried out a first ANOVA with factors Language Type (OV/VO) and Order (FI/IF) using the proportion of FI and IF “phrases” in the 10 subcorpora per language as the dependent measure. We obtained a significant main effect of Order [*F*_(1, 38)_ = 6.21, *p* = 0.017], as more IF than FI phrases were identified in the four languages. We also observed a significant main effect of Language Type [*F*_(1, 38)_ = 4.52, *p* = 0.040], because more phrases were identified in the VO than in the OV languages overall. Importantly, the interaction of Language Type and Order was very highly significant [*F*_(1, 38)_ = 60.56, *p* < 0.0001], because IF phrases abounded in OV, FI phrases in VO languages. In Scheffe *post-hoc* tests, the difference between the number of IF and FI phrases was significant in both OV (*p* < 0.0001) and VO languages (*p* < 0.001).

In a second ANOVA with factors Language (Basque/Japanese/French/Italian) and Order (FI/IF), we found a main effect of Order [*F*_(1, 36)_ = 6.06, *p* = 0.018] due to the greater number of IF phrases in the overall dataset, just like in the first ANOVA. Again, the interaction between Language and Order was significant [*F*_(1, 36)_ = 20.07, *p* < 0.0001]. In Scheffe *post-hoc* tests, we found significant differences between the number of IF and FI items in Basque (*p* < 0.0001) and in Japanese (*p* < 0.001), marginally so in Italian (*p* = 0.07) and not in French. Further, although numerically, the number of IF phrases was greater in Basque than in Japanese, this difference did not reach significance.

### Discussion

The analyses confirmed the general prediction that frequent items occupy sequence-initial positions in VO languages and sequence-final positions in OV languages. This distribution makes frequent items reliable cues to word order across typologically different languages. Through their distribution and sequential position, they can provide potential break-in points or anchors for the syntactic bracketing of sentential structure. This general pattern is modulated by language-specific properties. In the two OV languages, this pattern is stronger than in the VO languages. The reason for this difference between the OV and VO languages we tested is that while Japanese and Basque are functor-final both at the level of syntax (OV) and morphology (heavily suffixing), French and Italian are strongly functor-initial syntactically (VO), but have both suffixes and prefixes in their morphology.

In addition, we also observed an overall advantage for IF over FI items in all the languages. This effect was carried partly by the outstandingly high percentage of IF items in Basque, partly by the fact that the two VO languages had more IF items than the OV languages had FI items. The high percentage of IF sequences in Basque derives from the overt realization of the definite article phrase-finally and the generally strong functor-final nature of the language. (Japanese has no overt definite article.) Further, in the light of the discussion above, French and Italian are syntactically functor-initial, but somewhat more mixed morphologically, hence the non-negligible presence of IF phrases in these languages. More generally, this might reflect a universal preference for suffixation (Greenberg, 1957; Hawkins and Gilligan, 1988; Dryer, 1992). Languages with OV order almost exclusively use suffixation, i.e., word- and thus phrase-final morphological functors. However, many VO languages make use of suffixes in addition to prefixes. French and Italian are two cases in point.

As a caveat, we need to point out that the nature of the corpora differed somewhat between the languages. While most of the material in all four languages was derived from infant- and child-directed sources, the Basque corpus also contained some adult-directed material and was derived from written rather than spoken sources—due to the unavailability of infant- or child-directed spoken corpora in Basque. These differences might alter the results somewhat, although we do not expect the overall pattern of results to change considerably.

The corpus analysis has shown that the distribution and sequential position of the most frequent elements correlate with basic word order in the linguistic signal. As a second step in examining the cross-linguistic role of frequent items as predicted by the anchoring hypothesis, we now ask whether adult speakers of the above tested four languages are sensitive to the language-specific distributions of frequent items and whether they use this knowledge when parsing novel linguistic material.

## Study 2: An artificial grammar learning experiment

We tested the word order preferences of adult native speakers of Basque, Japanese, Italian, and French using an artificial grammar learning paradigm. If Basque and Japanese speakers have different preferences for the relative order of frequent and infrequent items than Italian and French speakers that provides cross-linguistic evidence for the anchoring or frequency-based learning mechanism, and suggests that it is operational throughout life.

### Materials and methods

#### Participants

Twelve adult native speakers of Basque (6 females; mean age: 27 years, range: 20–37), 20 monolingual adult native speakers of Italian (12 females; mean age: 24 years, range: 20–34), 20 monolingual adult native speakers of Japanese (16 females; mean age: 22 years, range: 20–28), and 20 monolingual adult native speakers of French (13 females; mean age: 23 years, range: 19–29) participated in the experiment for monetary compensation. Basque native speakers were also familiar with Spanish. To minimize the effects of Spanish, participants were included in the study if and only if they met all of the following criteria: (i) they were late learners of Spanish (= 4 years of age), (ii) their parents were native or native-like speakers of Basque (according to participants' self-report), (iii) they used Basque in their daily interactions with family and friends, (iv) they lived in a predominantly Basque-speaking area, and (v) Basque has been their dominant language all their lives. Participants reported no history of neurological, language, or hearing impairment.

#### Material

**Figure 2:**
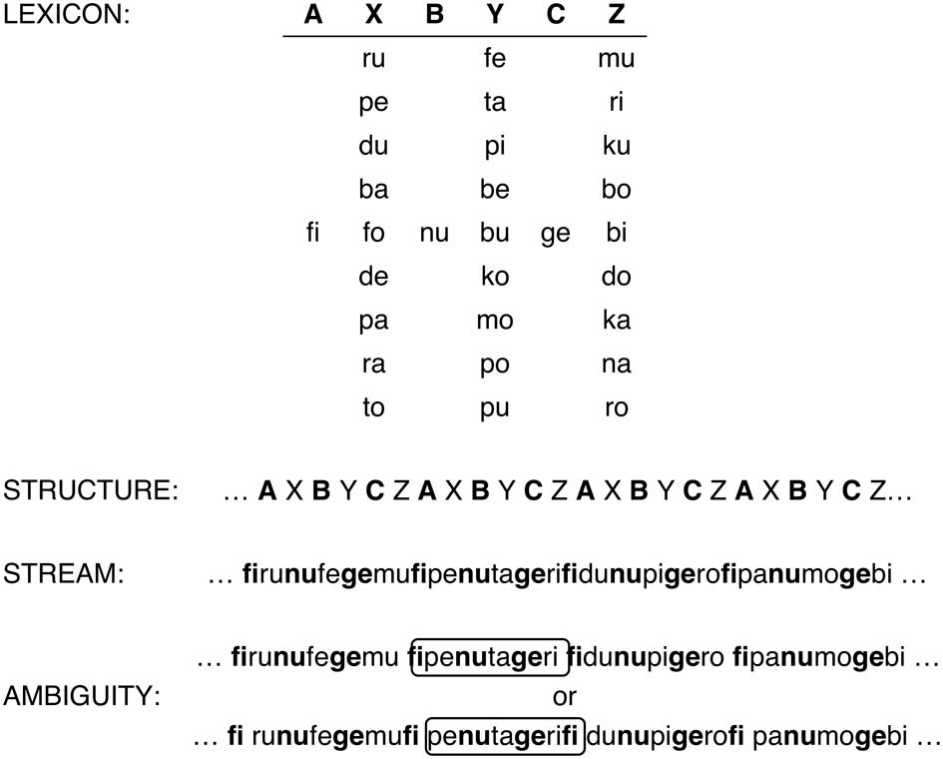
**The material used in the experiment**. The lexicon contains the syllables used in the three frequent and three infrequent categories. The structure shows how the six-syllable-long basic unit is concatenated to create the familiarization stream. The structural ambiguity is shown by the two possible parses of the stream. The upper tier illustrates the FI parse, the lower tier shows the IF one. The encircled chunks are examples of FI and IF test items, respectively.

A structurally ambiguous familiarization stream was created by repeatedly and continuously concatenating a six-syllable-long basic unit: AXBYCZ, where A, B, and C represented constant syllables, while X, Y, and Z came from three distinct categories of 9 syllables each (Figure 2). This way, the A, B, and C tokens were nine times more frequent than any particular syllable token from categories X, Y, and Z, mimicking the different frequency distributions of functors and content words, respectively. Since frequent and infrequent syllables followed each other in strict alternation, the stream was ambiguous between two possible parses: a FI order, i.e., AXBYCZ as the basic unit, or a IF order, i.e., XBYCZA as the basic unit (Figure 2). To ensure ambiguity, all phase information was eliminated by ramping the initial and final 15 s of the stream in amplitude. These manipulations created a continuous artificial grammar similar to the one used in Gervain et al. (2008), with the only difference that in the current study three rather than two frequent and infrequent categories were used in order to make the material more suitable for adults.

Care was taken to avoid phonotactic and frequency biases (i) by avoiding phoneme sequences that are illegal or infrequent in one or more of the languages and (ii) by matching the frequencies of syllables used in the initial positions of test items across the four languages^8^. All 36 syllables used to create the stream were chosen to have similar frequencies in word initial positions in Japanese, Italian, Basque, and French.

The familiarization stream was synthesized using the es1 Spanish male diphone database of MBROLA (Dutoit, 1997), with a monotonous pitch of 100 Hz and a constant phoneme duration of 120 ms. The six-syllabic basic unit was repeated 540 times, resulting in a 17 min 30 s long familiarization stream.

Test items were 36 six-syllable-long “sentences.” Half instantiated the FI order; the other half the frequent-final or IF order (see Figure 2 for an example). All frequent and infrequent categories were used with equal frequency in each position within test items. Infrequent tokens were used four times each to make up the 36 test items. Each test item was paired up with another that had the opposite word order to create 36 test pairs for the two-alternative forced choice (2AFC) paradigm. All test items were used twice; once as the first and another time as the second member of a test pair. The same test item never appeared in consecutive trials. The order of presentation was randomized and counterbalanced across participants.

#### Procedure

Participants were tested individually in sound-attenuated rooms. Sound stimuli were delivered through high-quality headphones. Participants were seated in front of a computer screen, where the instructions appeared. They were informed that they would first listen to a sample of an unknown language, and would then be tested on their knowledge of the “sentences” of the language. A short training session followed in order to familiarize participants with the 2AFC procedure, used later in the test phase. During training, participants heard 10 syllable pairs. The syllables were 11 consonant-vowel syllables, unrelated to the test items. For each pair, they had to identify a target syllable (“so”) by pressing one of two predefined keys depending on whether the target syllable appeared as the first or second member of the pair. After training, participants were instructed to listen to the familiarization stream, which lasted 17 min 30 s. After familiarization, participants passed immediately onto the test phase. In each of the 36 trials, they heard a pair of “sentences,” and they had to indicate by pressing one of two predefined keys which of the two “sentences” sounded more like a possible sentence of the unknown language. Items within a pair were separated by a pause of 500 ms.

The computer recorded the number of IF responses, which was used for data analysis. The number of FI responses can be obtained by subtracting this number from the total number of trials (36).

Basque participants were tested in the Psycholinguistics Laboratory of the University of the Basque Country, Vitoria-Gasteiz, Spain in collaboration with the Center for Brain and Cognition, Universitat Pompeu Fabra, Barcelona, Spain. Japanese participants were tested at Saitama University, Saitama, Japan. Italian participants were tested at the Language, Cognition and Development Laboratory of SISSA, Trieste, Italy. French participants were tested at the Cognitive Sciences and Psycholinguistics Laboratory, EHESS-ENS-CNRS, Paris, France.

### Results

**Figure 3:**
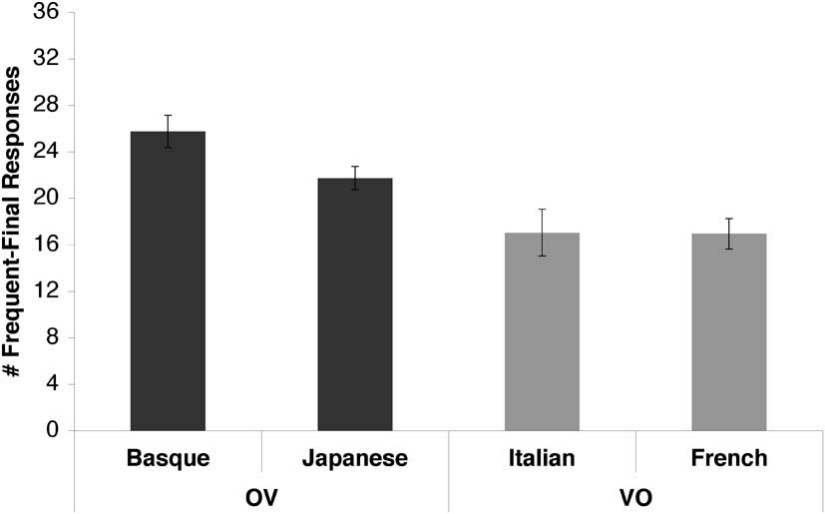
**Basque, Japanese, Italian, and French speakers' word order preferences**. The y-axis shows the number of IF responses given by the groups (out of a total of 36 test trials). Errors bars represent the standard errors of the mean. OV languages are shown in dark gray; VO languages in light gray.

Figure 3 shows the number of IF responses given by the participants. The average number of IF responses in the Basque group was 25.75 (±1.40 SE), which was significantly higher than chance [*t*_(11)_ = 5.54, *p* = 0.0002, *d* = 2.262]. The average number of IF responses in the Japanese group was 21.75 (±1.00 SE), which was also significantly higher than chance [*t*_(19)_ = 3.74, *p* = 0.001, *d* = 1.183]. In the Italian group, the average was 17.05 (±2.01 SE), which was not significantly lower than chance [*t*_(19)_ = −0.47, n.s.]. In the French group, the average was 16.95 (±1.31 SE), which was not significantly lower than chance [*t*_(19)_ = −0.80, n.s.].

We conducted a One-Way ANOVA with Language as a factor (Basque/Japanese/Italian/French) to allow for comparisons between languages. The main effect of language was significant [*F*_(3, 68)_ = 6.51, *p* = 0.0006, η_*p*2_ = 0.223], confirming that speakers of different languages had different preferences. This main effect was carried by significant differences between the following language pairs, as determined by *post-hoc* tests: Japanese and Italian (*p* = 0.024), Japanese and French (*p* = 0.021), Basque and Italian (*p* = 0.0005), Basque and French (*p* = 0.0004). The difference between Basque and Japanese showed a trend toward significance (*p* = 0.09), with Basque participants having a higher number of IF responses than Japanese participants. The difference between Italian and French was not significant.

When the results were pooled together in a more general comparison of the OV (Basque and Japanese) and VO (Italian and French) languages (Figure 3, dark gray vs. light gray bars), speakers of the two language types had highly significantly different word order preferences [*t*_(70)_ = 4.053, *p* = 0.0001, η_*p*2_ = 0.190], reflecting the fact that speakers of OV languages endorsed IF test items much more often.

### Discussion

The above results provide evidence that adult speakers of OV and VO languages have different preferences for the relative order of frequent and infrequent items. Strikingly, the respective word order preferences of the two populations followed very closely the statistical distributions of their respective native languages. Basque and Japanese participants preferred the IF order, which is characteristic of these languages. The strength of Basque speakers' preference for the IF order is particularly compelling, given the fact that they are familiar with Spanish, a VO, i.e., FI language. Italian and French participants' preference for the FI order did not reach significance, but it pointed in the expected direction. This asymmetry in OV and VO speakers' behavior is particularly interesting, as it mirrors the distributional data obtained in the corpus study. Just as Basque and Japanese are more systematically functor-final than French and Italian are functor-initial, so are speakers' preferences asymmetrical in exactly the same way. It thus seems that adult speakers' linguistic intuitions follow the statistical and distributional properties of their native languages quite closely, above and beyond large typological categories such as basic word order, which is also reflected in their behavior.

A language that is VO *and* prefixing, i.e., functor-initial both in syntax and morphology, would be the perfect equivalent to the OV languages tested in this study. However, these languages, e.g., some of the indigenous languages spoken in South America as well as some African languages, are harder to test in laboratory circumstances. The fact that Basque and Japanese participants, whose languages show a consistent functor-final order in syntax *and* morphology, have a significant preference in the expected direction, lends support to the above explanation. Even more interestingly, Basque speakers showed a somewhat stronger preference than Japanese participants, reflecting the difference in the distribution of IF items in the two languages. The graded nature of Japanese and Basque participants' preference thus further confirms the hypothesis that directionality both at the syntactic and morphological levels influences adult participants' responses.

## General discussion

In the current study, we investigated the cross-linguistic use of frequent items, i.e., functors, as anchor points for parsing novel linguistic material. First, we analyzed corpora from four languages, Japanese, Basque, French, and Italian, representing two different basic word orders, OV and VO. We found that the most frequent elements are indeed functors in all four languages and their sequential position strongly correlates with basic word order. Thus, in OV languages, frequent items are phrase-final, while in VO languages, they are phrase-initial. Second, we have shown that adult speakers of these languages are sensitive to the distributions of frequent items at utterance boundaries in their native language. They use them when parsing and organizing novel linguistic material, such as the artificial speech stream presented to them in our study. In other words, this rudimentary word order template is generalizable to novel speech input. We found this to be the case in languages that are historically, geographically, and genealogically unrelated.

In the broader perspective of language learning, we speculate that this frequency-based parsing mechanism offers one of the possible first steps in a cross-linguistically applicable account of how a rudimentary representation of basic word order might be established. We have shown that adults, like infants, are sensitive to the distributions of functors in key sentential positions. This simple, frequency-based knowledge could serve as a stepping-stone toward a more sophisticated knowledge of basic word order phenomena early in development. Future research will need to explore how this bootstrapping mechanism might take place. Minimally, what needs to be shown is that the categories established using frequency indeed map onto functors and content words. In addition, it must be shown that the relative position of functors and content words characterizing the input can be exploited to learn the relative positions of other syntactic categories (e.g., Heads, Complements, etc.), given the correlations that exist between the former order and the latter order. Some existing results with infants suggest that these logically necessary steps might be present already in early language acquisition. Hochmann et al. (2010) have shown that in an artificial grammar learning task similar to the one used here, but coupled with an object labeling task, 17-month-old infants were more likely to use the infrequent rather than the frequent words as object labels, suggesting that they expected infrequent words to have semantic content, as is typical of content words. Further, Bernard and Gervain (2012) have found that when prosodic prominence was added to the type of FI alternating stream used in the current study, 8-month-olds expected infrequent items to be prosodically prominent, as is typical of content words, and frequent items not to carry prosodic prominence, as is usually the case of functors. Thus, frequency-based categories, similar to the ones established in the current study, carry the hallmarks of broad lexical categories in natural language already during the first years of language development. Another suggestive finding indicates that 8-month-old infants expect frequency-based word order representations similar to the ones used in the current study to carry the phrasal properties typical of OV and VO phrases in natural languages (Gervain and Werker, 2013). Bilingual infants exposed to an OV and a VO language chose the frequent-initial parse of the artificial grammar when they heard the stream with a durational-contrast based prosody, typical of VO languages (Nespor et al., 2008; Shukla and Nespor, 2010). But they preferred the frequent-final parse when exposed to a pitch-contrast based prosody, found in OV languages. More research will be needed in the future to confirm these hypotheses.

One particularly interesting aspect of our results becomes obvious when we compare our behavioral findings with those of the analogous infant study (Gervain et al., 2008). Similarly to infants, adults also use frequent functors as anchor point. This suggests that some parsing mechanisms might be continuous throughout life: frequent elements indicate key sequential positions (onset/end). These peripheral positions might be relevant for language processing throughout development because they have been shown to be encoded and remembered better than other sequential positions, e.g., sequence-middles (Braine, 1963a,b, 1966; Henson, 1998; Ng and Maybery, 2002; Endress et al., 2009). However, there is one key difference between infants' and adults' behavior. Young learners in the Gervain et al. (2008) study did not show the same OV-VO asymmetry as adults in the current study do. Both Japanese and Italian infants showed a clear preference for the order of their respective native language, whereas for adults the preference is much more pronounced in the Japanese and the Basque groups than in the French and Italian ones. One possible explanation for this difference is that adults might follow the distributional properties of their native language more closely than infants do. As adults have more experience with their native language *and* have much greater memory capacity than infants, they are able to track the distributions of specific items and item categories with precision. Given infants' more limited experience as well as smaller memory capacity, their best processing strategy might be to extract overarching regularities. This explanation converges with previous experimental findings regarding the differences between adults' and infants' processing and learning strategies (e.g., Hudson Kam and Newport, 2009) and converges well with the “less is more” hypothesis (Newport, 1990) linking infants' typical (over)regularization behavior to their more limited memory capacity.

Notice that the frequency-based learning strategy, like many other such mechanisms, is necessarily a heuristic method. It provides the learner with a general word order pattern for the target language. Word order, however, is not always consistent across different phrase types within a language. While most languages, like Basque or Italian, follow the basic word order in all phrases, other languages, like German or Dutch, allow different orders even within the same phrase type. German, for example, has both OV and VO orders in verb phrases (*Anna trinkt Wasser* Anna drink.3sg water “Anna drinks water.”, but *Anna hat Wasser getrunken* Anna have.3sg water drunk “Anna has drunk water.”). The frequency-based learning strategy cannot capture these cases. We suggest that what it provides is an initial, general, and rudimentary representation of word order. Other learning mechanisms, such as prosodic bootstrapping (Nespor et al., 1996, 2008; see also Langus and Nespor, 2010), might complement this representation and derive the different word order patterns that occur in a language. Speakers of mixed languages might, therefore, be able to exploit other cues in addition to frequency, a prediction that will require further research.

## Conclusion

Understanding the learning mechanisms that account for how morphosytactic properties might be acquired across different languages is crucial to any theory of language, as language-specific knowledge is initially not available to young learners. However, few of the mechanisms proposed in the literature have been tested cross-linguistically. Here, we show that a frequency-based account of the sentential position of functors holds across typologically different languages. We also present evidence that this learning mechanism is used by young as well as mature language learners when facing novel linguistic material, but the underlying processing and learning mechanisms might be somewhat different in the two populations. Adults might rely more heavily on statistical and distributional information, while infants might generalize and extract regularities.

These results confirm the hypothesis according to which one universal role of frequent functional items is to contribute to the learnability of language by signaling the boundaries of syntactic units.

### Conflict of interest statement

The authors declare that the research was conducted in the absence of any commercial or financial relationships that could be construed as a potential conflict of interest.

## Footnotes

^1^Italian: *sul tavolo on*-the table “on the table,” where *sul is* more frequent than *tavolo;* Japanese *Kobe* ni Kobe to “to Kobe,” where *ni is* more frequent than *Kobe*.

^2^Children at the two-word stage also produce a large number of utterances that only contain content words, typically noun–noun combinations. Indeed, the production of noun–noun utterances at this early stage is correlated with higher vocabularies and general language development (Goldin-Meadow et al., 1976).

^3^ Grammatical markers are the most frequent means by which languages meet parsing and processing constraints. However, others, like rigid word order, also exist. Indeed, languages may differ in the extent to which they make use of these grammatical tools. Heavily agglutinating/morphologically rich languages like Basque or Turkish represent one possibility, i.e. the extensive use of markers, whereas Chinese, with only a handful of grammatical functors, but very rigid word order, embodies another option.

^4^The rationale of the current corpus study is similar to the one in Gervain et al. (2008). Some details of the methodology differ between the two studies, most importantly the use of morpheme instead of word counts, hence the numerical differences in results. Importantly, we take no position as to the free/bound status of these morphemes from a theoretical point of view, as our primary focus is to test a bootstrapping heuristic, i.e. a mechanism of surface parsing.

^5^However, two- and three-word utterances were kept, and contributed two data points (beginning and end) to the total, in the same way as longer sentences did. In the case of two-word utterances, this means that the same IF or FI phrase counted twice. This, however, is not a problem, as we are interested in the *proportion* of IF and FI utterances with respect to the total and not their absolute numbers. Because the number of utterances is constant across samples and languages, and the number of two-word utterances is not different across languages, whether they count as one or two data points doesn't change their relative contribution.

^6^Other thresholds (0.005, 0.0025, and 0.001) were also tested. As they provided very similar results, they will not be reported separately here.

^7^This is not to say that other patterns, e.g., frequent items utterance-initially and infrequent items utterance-finally or vice versa, might not be informative about word order. We chose the FI and IF sequences at utterance boundaries, because they are simple, locally identifiable patterns.

^8^Syllable frequencies were taken from standard frequency dictionaries for Italian and French (http://www.lexique.org/), and were kindly provided by The Basque Language Institute for Basque and by RIKEN for Japanese.

## Appendix

### List of test items

IF items:

  begebifibanu

  bifiranupoge

  bofipanumoge

  bugemufipanu

  dofirunubege

  dunupugekufi

  fegekafibanu

  fegerifirunu

  kogenafipenu

  kufibanupige

  mufifonupuge

  panukogemufi

  penubegedufi

  pigekufipanu

  rofirunumoge

  runupugerifi

  tagekafiranu

  tonukogerifi

  *FI items:*

  fibanutagebi

  fidunupigena

  fipanumogeku

  fitonubegero

  gebifiranupu

  gebofifonumo

  gekafibanube

  gekufipanufe

  gemufidunupi

  genafiranuta

  gerofiranubu

  nubegebifiba

  nufegemufifo

  nukogebofiru

  nukogekafide

  nupigerifira

  nupigerofiba

  nupugedofiru
